# Family-based exome sequencing identifies candidate genes related to keratoconus in Chinese families

**DOI:** 10.3389/fgene.2022.988620

**Published:** 2022-09-02

**Authors:** Liyan Xu, Kaili Yang, Shanshan Yin, Yuwei Gu, Qi Fan, Yawen Wang, Dongqing Zhao, Shengwei Ren

**Affiliations:** ^1^ Henan Provincial People’s Hospital, Henan Eye Hospital, Henan Eye Institute, People’s Hospital of Zhengzhou University, Henan University People’s Hospital, Zhengzhou, China; ^2^ Zhengzhou University People’s Hospital, Henan Provincial People’s Hospital, Henan Eye Hospital, Henan Eye Institution, Zhengzhou, China; ^3^ Henan University People’s Hospital, Henan Provincial People’s Hospital, Henan Eye Hospital, Henan Eye Institute, Zhengzhou, China

**Keywords:** keratoconus, family-based exome sequencing, candidate genes, genetic etiology, bioinformatics analysis

## Abstract

**Background:** Keratoconus (KC) is a complex corneal disorder with a strong genetic component. The present study aimed to identify candidate genes related to KC in Chinese families.

**Methods:** Family-based exome sequencing was performed in ten patients suffering from KC who belong to five families with two affected members in each. The candidate rare variants were identified with multi-step bioinformatics analysis. The STRING website was used to perform the protein interaction of the identified genes.

**Results:** Our analyses identified 32 candidate rare variants in 13 genes by family-based exome sequencing. The molecular analyses of identified genes showed that EPCAM directly interacted with CTNNB1 of the Hippo signaling pathway and focal adhesion pathway, and directly interacted with CTNNB1, CDH1 of the WNT signaling pathway. SHROOM3 directly interacted with ROCK2, ROCK1 of the focal adhesion pathway. SYNE1 directly interacted with MUSK of the extracellular matrix organization pathway. TEK directly interacted with VEGFA, SHC1, PIK3R1, GRB2 of the focal adhesion pathway. TTN directly interacted with CAPN3 of the extracellular matrix organization pathway.

**Conclusion:** The *EPCAM*, *SHROOM3*, *SYNE1*, *TEK*, and *TTN* genes were potential high-risk candidate pathogenic genes of familial KC. The findings might significantly improve our understanding of the genetic etiology of the disease, providing novel insights on KC pathogenesis.

## Introduction

Keratoconus (KC) is characterized by progressive corneal protrusion and thinning, leading to irregular astigmatism and impairment of visual function (Rabinowitz, 1998). The estimated prevalence of KC in the whole population is 1.38 per 1,000 people (Hashemi et al., 2020). The disease usually begins at puberty and progresses into the third or fourth decades (Santodomingo-Rubido et al., 2022). Currently, no curative treatments are available for KC (Mohammadpour et al., 2018). The progressive corneal thinning can be stabilized with corneal cross-linking when it is recognized at an early stage (Gomes et al., 2015). However, corneal transplantation is necessary for advanced cases (Sarezky et al., 2017). Thus, an early diagnosis of KC is crucial for improving its prognosis. Notably, understanding the pathogenesis of KC could help in the achievement of an early diagnosis and timely treatment of the disease.

KC is considered as a complex corneal disorder determined by a combination of environmental and genetic factors (Lucas and Burdon, 2020). Environmental factors included eye rubbing, allergies, diabetes, and sleeping position, as highlighted in previous studies (Ahuja et al., 2020; Mazharian et al., 2020; Ates et al., 2021). The higher concordance rate in monozygotic twins (Tuft et al., 2012), and a positive family history of 5%–23% in KC cases (Rabinowitz et al., 2021) suggested a strong genetic component in the development of KC. Indeed, many researchers have identified KC susceptibility genes by genome-wide association studies (Hosoda et al., 2020; Hardcastle et al., 2021), linkage studies (Hughes et al., 2011; Karolak et al., 2015) and candidate gene sequencing analyses (Abdelghany et al., 2021; Lopes et al., 2021). The genetic studies on KC significantly contributed to the biological basis of its pathogenesis. However, the genetic basis of KC susceptibility has not been fully understood due to the genetic heterogeneity and population differences, and the pathogenesis underlying the genetic variants remains unclear.

Currently, several genetic studies have been performed on KC in Chinese populations, and identified some genetic variants accounted for the disease (Hao et al., 2017; Xu et al., 2020; Zhang et al., 2020; Lin et al., 2022; Yuan et al., 2022). Nevertheless, the majority of the studies were performed in sporadic cases or one pedigree. The complex etiology of KC with a strong genetic heterogeneity still needs to be fully elucidated. Thus, the aim of this study was to identify candidate genes potentially related to KC predisposition in families with KC. Consequently, family-based exome sequencing of ten patients with KC from five Chinese families were performed in the present study, and bioinformatics approaches were used to determine the genetic factors contributing to the onset of the disease.

## Materials and methods

### Family recruitment

A total of ten patients with KC from five families with two affected members in each were selected for the current study. The diagnosis of KC was based on clinical manifestations such as localized stromal thinning, conical protrusion, Vogt’s striae, Fleischer’s ring, or anterior stromal scar, as well as signs of corneal topography revealing an asymmetric bowtie pattern with or without skewed axes (Mas Tur et al., 2017). Patients whose KC was caused by trauma, other disease, or surgery were excluded from the study. The study was approved by the Institutional Review Board of Henan Eye Hospital [ethical approval number: HNEECKY-2019(5) and performed in accordance with the guidelines of the Declaration of Helsinki. Written informed consent was obtained from each subject.

### Exome sequencing

Total genomic DNA was extracted from peripheral blood samples according to the manufacturer’s recommendations. DNA quality was examined by Qubit 3.0 and confirmed by electrophoresis prior to library construction. The DNA was fragmented to an average size of 180–280 bp and subjected to DNA library creation with established Illumina paired-end protocols. The library was then subjected to exome sequence capture by Agilent SureSelect Human All ExonV6 Kit (Agilent Technologies, Santa Clara, CA, United States) according to the manufacturer’s instructions. The Illumina Novaseq 6000 platform (Illumina Inc., San Diego, CA, United States) was used for genomic DNA sequencing in Novogene Bioinformatics Technology Co., Ltd. (Beijing, China).

### Bioinformatics analysis

**FIGURE 1:**
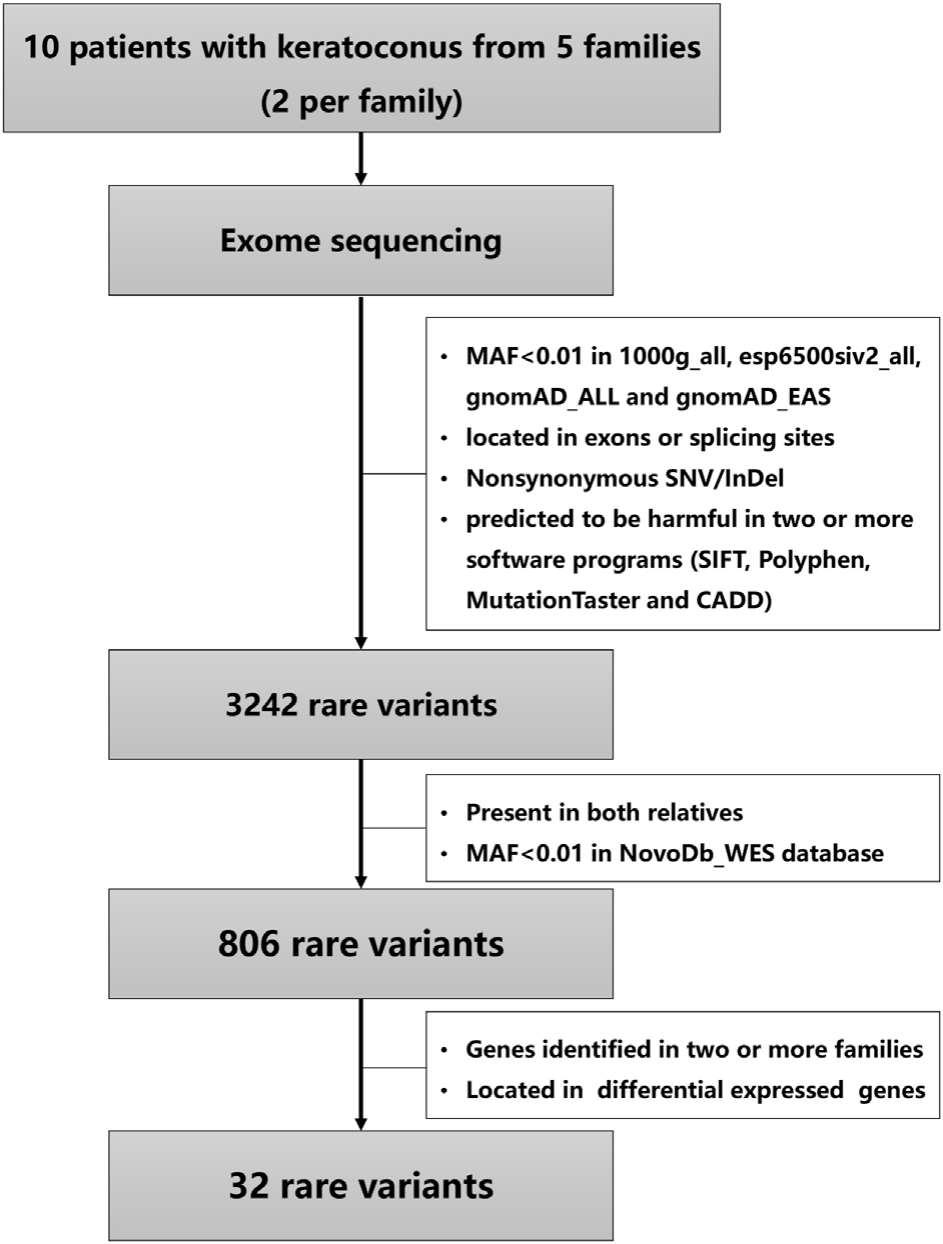
An overview of stepwise filtering of variants identified in keratoconus families.

After quality control, the sequencing reads were mapped to hg19 (GRCh37) using Burrows-Wheeler Aligner (Li and Durbin, 2009), and duplicate reads were marked using Sambamba tools (Tarasov et al., 2015). SAMtools (Li et al., 2009) were used to perform variant calling to identify single nucleotide variants (SNVs) and small insertions or deletions (InDels). The raw calls of SNVs and InDels were further filtered with the following inclusion thresholds: (1) read depth >4; (2) Root-Mean-Square mapping quality of covering reads >30; (3) variant quality score >20. Then annotation of the variants was performed using ANNOVAR (Wang et al., 2010). As is shown in Figure 1, the variants were firstly filtered using the following criteria: (1) variants with a minor allele frequency less than 0.01 in 1,000 genomic data (1000 g_all), esp6500siv2_all, and gnomAD data (gnomAD_ALL and gnomAD_EAS); (2) variants located in exons or splicing sites; (3) variants predicted to influence splicing or amino acid alternation; (4) variants predicted to be harmful in more than half of the software programs (SIFT, Polyphen, MutationTaster and CADD) according to the scores. Secondly, the variants presented in both relatives and variants with a minor allele frequency less than 0.01 in NovoDb_WES database (2,573 healthy Chinese control individuals) were selected. Finally, the variants located in genes presented in two or more families and differentially expressed in KC were considered as candidate rare variants (Lee et al., 2009; Mace et al., 2011; Bykhovskaya et al., 2016; Kabza et al., 2017; Khaled et al., 2018; You et al., 2018; Sharif et al., 2019; Shinde et al., 2020; Sun et al., 2022). The STRING website was used to predict the relationships of proteins with previously reported genes in KC and known KC-associated pathways, including extracellular matrix organization, WNT signaling, Hippo signaling, focal adhesion and TGF-β pathways (Cai et al., 2020; Hao et al., 2021).

## Results

### Clinical characteristics of patients with Keratoconus

**FIGURE 2:**
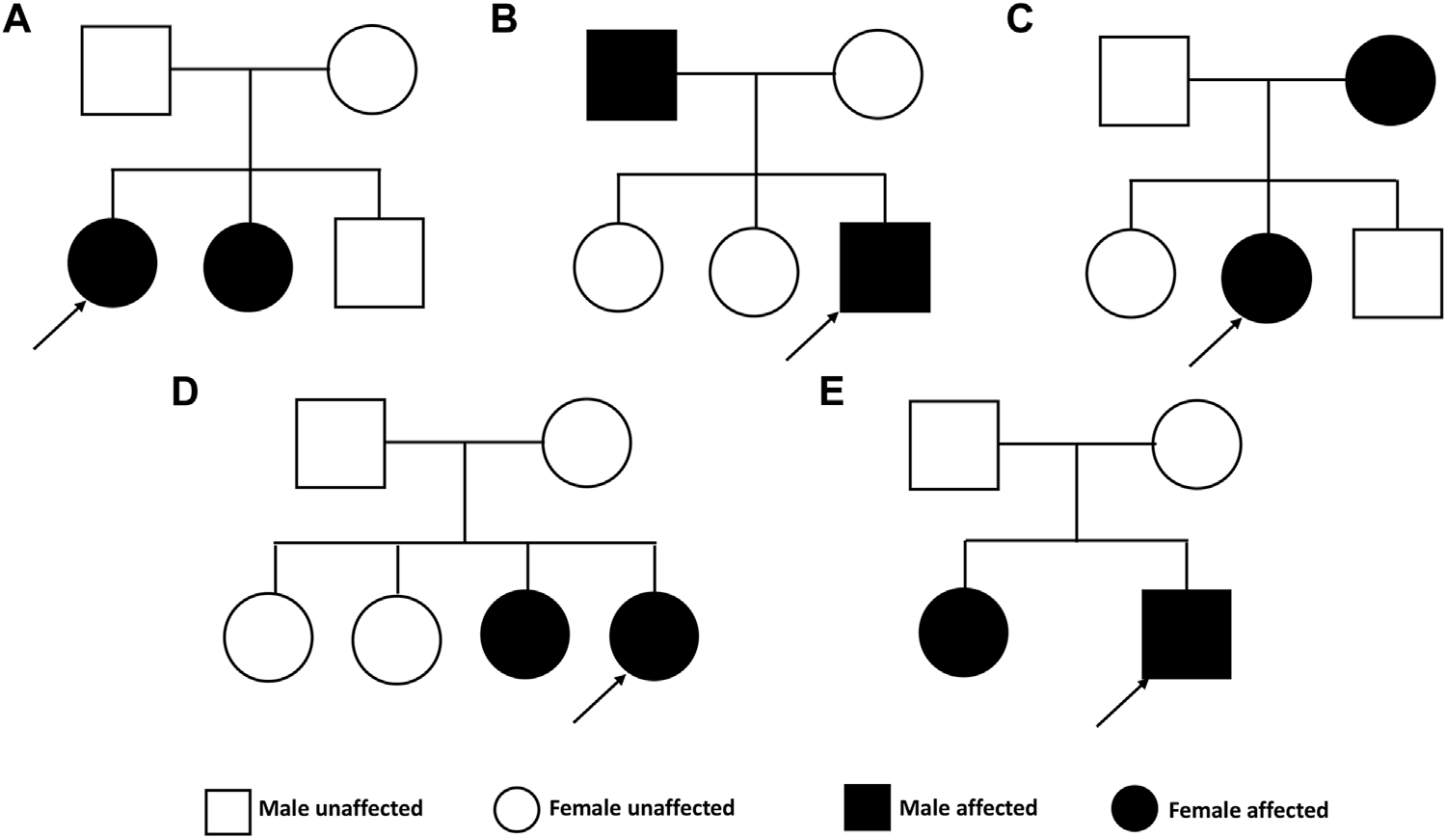
Pedigrees of the Chinese family with keratoconus.

The pedigrees of the five investigated families are presented in Figure 2. The clinical characteristics of the ten patients with KC are listed in Table 1. The mean age at diagnosis was 28 years (range from 18 to 54). In this study, three patients were male and seven patients were female. The clinical investigation revealed the presence of Vogt’s striae in one patient, Munson’s sign in four patients, and Fleischer’s ring in seven patients.

**TABLE 1:** Clinical characteristics of keratoconus families.

Family ID	Individual ID	Sex	Age at diagnosis	Mean keratometry (D)		Thinnest corneal thickness (μm)		Vogt’s striae	Fleischer’s ring	Munson’s sign
				OD	OS	OD	OS			
Family A	Proband	F	24	40.0	74.2	436	234	OU−	OU+	OS+
	Sister	F	18	43.0	43.7	482	506	OU−	OU+	OU−
Family B	Proband	M	24	46.9	47.4	404	406	OU−	OU+	OU+
	Father	M	54	44.4	44.4	451	461	OU−	OU+	OU−
Family C	Proband	F	23	54.8	55.0	382	398	OD+	OU+	OD+
	Mother	F	54	45.5	46.7	438	422	OU−	OU−	OU−
Family D	Proband	F	18	49.9	61.5	420	360	OU−	OU+	OU+
	Sister	F	20	44.6	45.8	461	453	OU−	OU−	OU−
Family E	Proband	M	18	43.5	47.8	516	476	OU−	OS+	OU−
	Sister	F	27	44.1	45.8	515	517	OU−	OU−	OU−

### Candidate rare variants identified in Keratoconus families

The variants were filtered to screen the candidate rare variants in KC families according to the analysis workflow in Figure 1. Firstly, the variants focusing on frequency, location, and effects of the variants were filtered. Consequently, a total of 3,242 rare variants were identified. The screening of the variants that occurred in both the affected relatives with a minor allele frequency less than 0.01 in NovoDb_WES database identified 806 rare variants. Then the variants located in genes presented in two or more families and differentially expressed in KC were selected as candidate rare variants. Finally, 32 variants in 13 genes were identified, including 28 missense variants, one nonframeshift deletion variants, one stop gained variant, and two splicing variants (Table 2). Among the identified genes, the dynein axonemal heavy chain 6 (*DNAH6*), epithelial cell adhesion molecule (*EPCAM*), and titin (*TTN*) were reported to be upregulated in KC (Kabza et al., 2017). The ATPase H+ transporting V0 subunit e2 (*ATP6V0E2*), dynein axonemal heavy chain 5 (*DNAH5*), phosphodiesterase 11A (*PDE11A*), spectrin repeat containing nuclear envelope protein 1 (*SYNE1*), TEK receptor tyrosine kinase (*TEK*), TRIO and F-actin binding protein (*TRIOP*), and tyrosinase related protein 1 (*TYRP1*) genes were reported to be downregulated (Kabza et al., 2017; You et al., 2018; Shinde et al., 2020). However, the expressions of protocadherin beta 7 (*PCDHB7*), shroom family member 3 (*SHROOM3*), and WD Repeat Domain 81 (*WDR81*) genes exhibited inconsistent results in different studies (Kabza et al., 2017; You et al., 2018; Sharif et al., 2019; Shinde et al., 2020; Sun et al., 2022). In addition, the candidate variants in TTN were identified in three families (60%), while variants in other genes were identified in two families (40%).

**TABLE 2:** Candidate rare variants identified by exome sequencing in the five keratoconus families.

Family ID	Gene	Transcript	cDNA change	AA change	Function
Family A	*ATP6V0E2*	NM_001100592	c.C263T	p.T88I	Missense SNV
	*TTN*	NM_003319	c.G48646A	p.A16216T	Missense SNV
	*TRIOBP*	NM_001039141	c.1612_1614del	p.538_538del	Nonframeshift deletion
	*TYRP1*	NM_000550	c.A212G	p.D71G	Missense SNV
	*WDR81*	NM_001163809	c.A2866C	p.K956Q	Missense SNV
Family B	*DNAH5*	NM_001369	c.G12883A	p.V4295M	Missense SNV
	*DNAH5*	NM_001369	c.975 + 6C > T	—	Splicing
	*PDE11A*	NM_001077196	c.T208C	p.C70R	Missense SNV
	*SHROOM3*	NM_020859	c.A4726G	p.K1576E	Missense SNV
	*SYNE1*	NM_033071	c.A13556G	p.N4519S	Missense SNV
	*TEK*	NM_001290078	c.A949C	p.N317H	Missense SNV
Family C	*DNAH6*	NM_001370	c.G4358A	p.R1453H	Missense SNV
	*EPCAM*	NM_002354	c.G458C	p.R153T	Missense SNV
	*PDE11A*	NM_001077196	c.T935A	p.L312Q	Missense SNV
	*SHROOM3*	NM_020859	c.G1397A	p.S466N	Missense SNV
	*SHROOM3*	NM_020859	c.C3731T	p.P1244L	Missense SNV
	*SYNE1*	NM_033071	c.C4744T	p.L1582F	Missense SNV
	*TRIOBP*	NM_001039141	c.C4726T	p.R1576C	Missense SNV
Family D	*ATP6V0E2*	NM_001100592	c.A388C	p.S130R	Missense SNV
	*DNAH5*	NM_001369	c.A11735G	p.H3912R	Missense SNV
	*EPCAM*	NM_002354	c.G131A	p.R44H	Missense SNV
	*PCDHB7*	NM_018940	c.G1894T	p.E632X	Stopgain
	*TEK*	NM_001290078	c.G1787T	p.G596V	Missense SNV
	*TTN*	NM_003319	c.T72341C	p.V24114A	Missense SNV
	*WDR81*	NM_001163809	c.G3211A	p.V1071I	Missense SNV
Family E	*DNAH6*	NM_001370	c.C6655T	p.R2219C	Missense SNV
	*PCDHB7*	NM_018940	c.G1280T	p.G427V	Missense SNV
	*TTN*	NM_001256850	c.32791 + 2T > C	—	Splicing
	*TTN*	NM_001256850	c.T23805G	p.D7935E	Missense SNV
	*TTN*	NM_133379	c.C16631T	p.T5544M	Missense SNV
	*TTN*	NM_001256850	c.A1640G	p.Q547R	Missense SNV
	*TYRP1*	NM_000550	c.C785T	p.T262M	Missense SNV

### Molecular analysis of the identified genes

**FIGURE 3:**
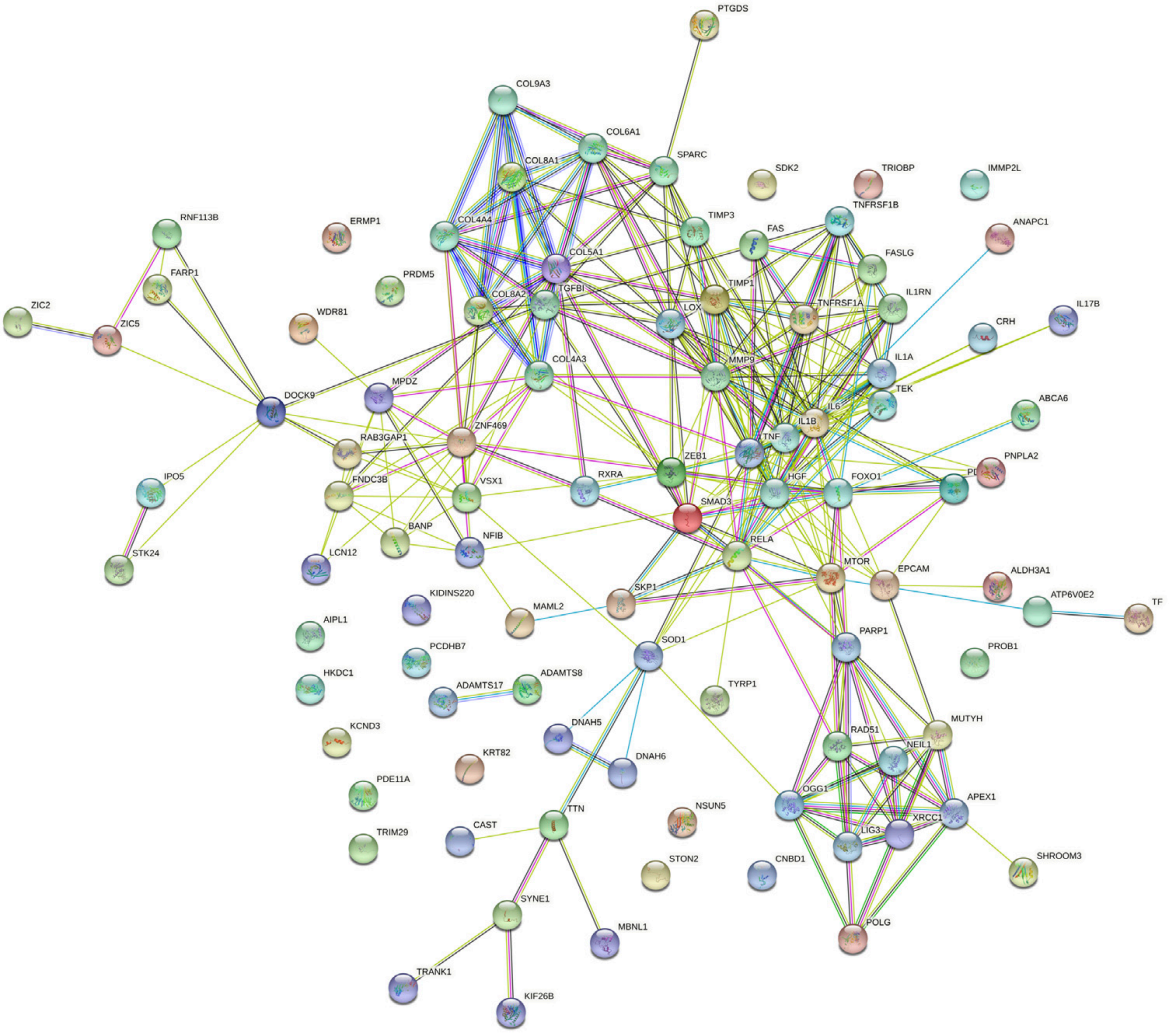
Protein-to-protein interactions of identified thirteen candidate genes with previously reported genes in keratoconus.

**FIGURE 4:**
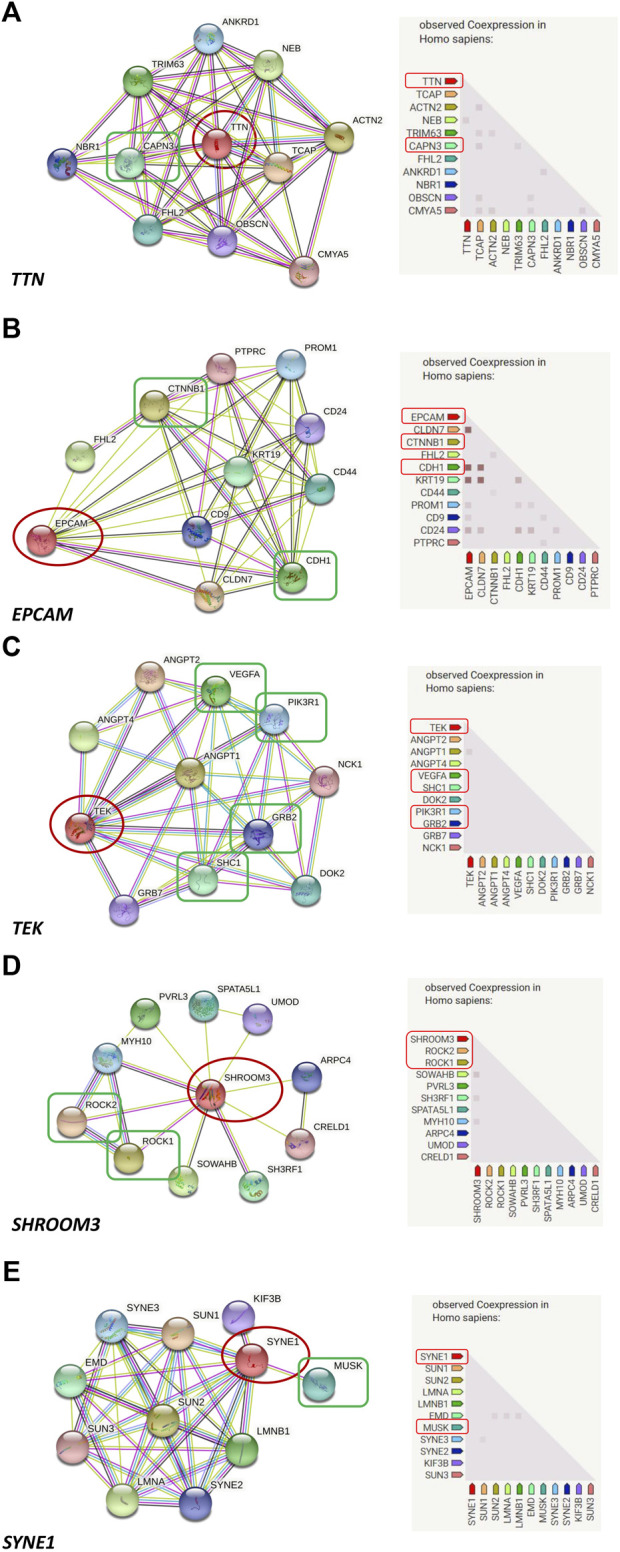
Protein-to-protein interactions of candidate genes with known keratoconus associated pathways. (**(A)**: EPCAM directly interacts with CTNNB1 of the Hippo signaling and Focal adhesion pathways, and directly interacts with CTNNB1, CDH1 of the WNT signaling pathway; **(B)**: SHROOM3 directly interacts with ROCK2, ROCK1 of the Focal adhesion pathway; **(C)**: SYNE1 directly interacts with MUSK of the extracellular matrix organization pathway; **(D)**: TEK directly interacts with VEGFA, SHC1, PIK3R1, GRB2 of the Focal adhesion pathway; **(E)**: TTN directly interacts with CAPN3 of the extracellular matrix organization pathway).

**FIGURE 5:**
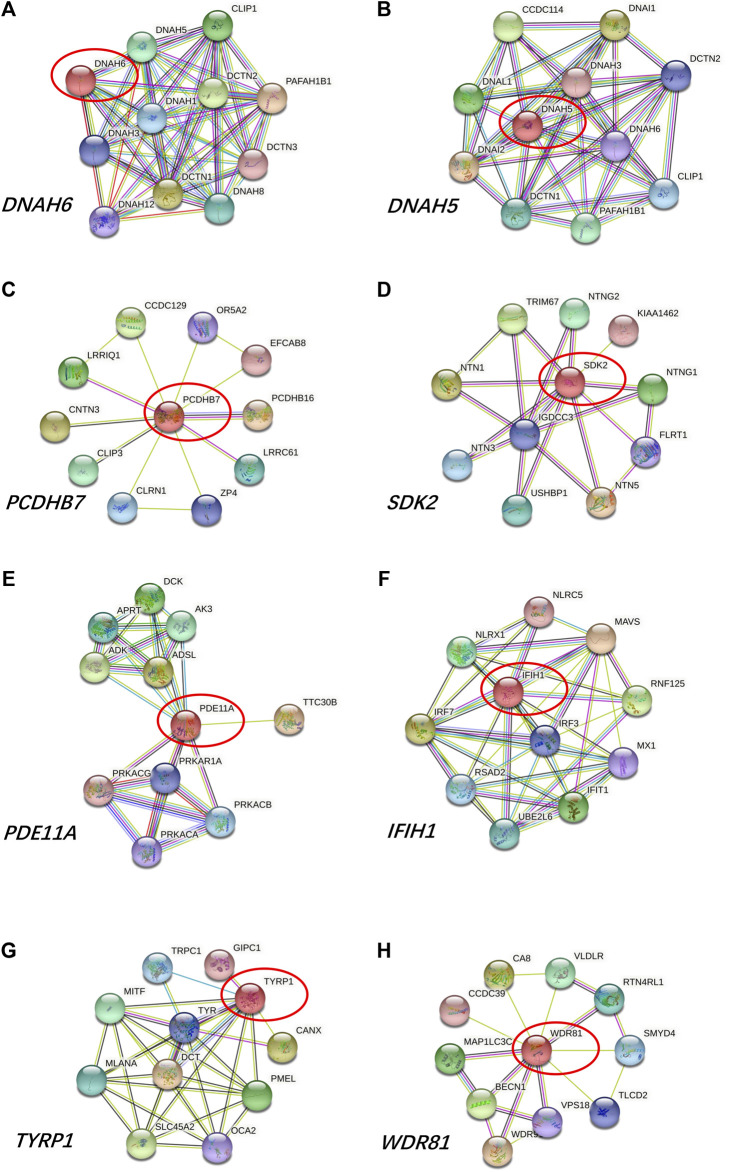
Protein-to-protein interactions of **(A)** *ATP6V0E2*, **(B)** *DNAH5*, **(C)** *DNAH6*, **(D)** *PCDHB7*, **(E)** *PDE11A*, **(F)** *TRIOBP*, **(G)** *TYRP1*, and **(H)** *WDR81* genes.

Prediction of protein-protein interactions of the thirteen genes (*ATP6V0E2*, *DNAH5*, *DNAH6*, *EPCAM*, *PCDHB7*, *PDE11A*, *SHROOM3*, *SYNE1*, *TEK*, *TRIOBP*, *TTN*, *TYRP1*, and *WDR81*) was conducted using the online STRING software. The protein-protein interactions of the thirteen genes with previously reported 88 genes in KC were listed in Figure 3. The results showed that the genes directly interact with previously reported genes such as *SOD1*, *CAST*. Besides, the protein-protein interactions of identified genes with five KC-associated pathways (extracellular matrix organization, WNT signaling, Hippo signaling, focal adhesion and TGF-β pathways) were also analyzed. According to the interaction network shown in Figure 4, EPCAM directly interacted with CTNNB1 of the Hippo signaling and focal adhesion pathways, and directly interacted with CTNNB1, CDH1 of the WNT signaling pathway. SHROOM3 directly interacted with ROCK2, ROCK1 of the focal adhesion pathway. SYNE1 directly interacted with MUSK of the extracellular matrix organization pathway. TEK directly interacted with VEGFA, SHC1, PIK3R1, GRB2 of the focal adhesion pathway. TTN directly interacted with CAPN3 of the extracellular matrix organization pathway. However, other eight genes did not show any interaction with the investigated pathways (Figure 5). Our results highlighted that *EPCAM*, *SHROOM3*, *SYNE1*, *TEK*, and *TTN* were potential high-risk candidate pathogenic genes of KC that exert their effects by the disruption of the extracellular matrix organization, WNT signaling, Hippo signaling, focal adhesion and TGF-β pathways.

## Discussion

KC is a complex disease, with numerous genetic and environmental factors potentially involved in its pathogenesis (Lucas and Burdon, 2020). Although multiple studies on the etiology of KC have been performed, no consensus has been reached to date. In the present study, a family-based exome sequencing in five Chinese KC families was performed, with the aim to identify potential candidate genes contributing to KC susceptibility. By applying several filtering strategies, we identified 32 variants located in *ATP6V0E2*, *DNAH5*, *DNAH6*, *EPCAM*, *PCDHB7*, *PDE11A*, *SHROOM3*, *SYNE1*, *TEK*, *TRIOBP*, *TTN*, *TYRP1*, and *WDR81* genes as candidate rare variants. Bioinformatics analysis revealed that the *EPCAM*, *SHROOM3*, *SYNE1*, *TEK*, and *TTN* genes were potential high-risk candidate pathogenic genes of KC because of their relationships with known KC-associated pathways.

Genetic factors are implicated in the pathogenesis of KC, and multiple studies have identified numerous loci that might contribute to KC by high-throughput sequencing (Hosoda et al., 2020; Xu et al., 2020; Hardcastle et al., 2021). Although some genetic studies on KC families have been performed, and identified several candidate genes for KC (Froukh et al., 2020; Shinde et al., 2021), the family-based exome sequencing in Chinese KC families is limited. Our present study performed a family-based exome sequencing in five Chinese KC families. A total of 32 candidate rare variants located in 13 genes were finally identified by a series of filtering steps. Protein-protein interactions of the thirteen genes with previously reported genes in KC showed direct or indirect interaction with previously reported genes, indicating potential associations with KC. In addition, the *EPCAM*, *SHROOM3*, *SYNE1*, *TEK*, and *TTN* genes were considered as potential high-risk candidate pathogenic genes in KC after analyzing the protein-protein interactions of the thirteen genes with known KC-associated pathways.

EPCAM is a cell surface molecule involved in cell-to-cell adhesion, and plays significant roles in the modulation of proliferation, differentiation, and migration of epithelial cells (Huang et al., 2018). The molecular analysis of EPCAM indicated its interactions with CTNNB1 and CDH1 which are involved in WNT signaling, Hippo signaling, and focal adhesion pathways, suggesting a potential role of EPCAM in the pathogenesis of KC. SHROOM3 directly interacted with proteins involved in the focal adhesion pathway, and is a central regulator of morphogenetic cell shape changes in certain tissues (Tariq et al., 2011). As far as we know, KC is a bilateral and usually asymmetrical disease in which the ectatic cornea becomes conical in shape. Therefore, we speculated that the SHROOM3 gene might play roles in the pathogenesis of KC because of its interaction with proteins of the focal adhesion pathway and its potential functions in regulating cell shape. The SYNE1 gene encodes nesprin-1, a scaffold protein associated with anchoring the plasma membrane to the actin cytoskeleton and involved in the binding between the cytoskeleton, nuclear envelope and other subcellular compartments (Swan et al., 2018). Moreover, SYNE1 interacted with MUSK which is a gene involved in the extracellular matrix organization pathway, indicating a relationship between SYNE1 and KC. The TEK gene encodes a tyrosine kinase receptor and plays a central role in vascular stability (Gal et al., 2020). The gene directly interacted with VEGFA, SHC1, PIK3R1, GRB2 of the focal adhesion pathway, as revealed by the molecular analysis, which was associated with the pathogenesis of KC. Mutations in TEK might result in pathogenic effects by disrupting the focal adhesion pathway, leading to KC. Titin (TTN) is the largest protein in the human body, which is encoded by 364 exons of the *TTN* gene. It is reported that the TTN protein plays important roles in the regulation of the cytoskeleton organization in cardiomyocyte (Loescher et al., 2021). Among its interaction proteins, CAPN3 is involved in the extracellular matrix organization which is identified as a related pathway with KC. Thus, we speculated that *TTN* might be considered as a candidate gene for KC due to its indirect interaction with the extracellular matrix organization. In addition, TTN is considered as a major determinant of cardiomyocyte stiffness, and mutations in *TTN* might result in dilated cardiomyopathy in which myocardial stiffness has an important role in its pathogenesis (Begay et al., 2015). KC is a corneal disorder with its corneal stiffness changed. Mutations in *TTN* were both existed and predicted to be causative in dilated cardiomyopathy and KC, indicating that there might some similar molecular mechanism between them. Although no studies reported mutations in the identified six genes in KC, the genes might be involved in the pathogenesis of KC through their indirect interactions with the known KC-associated pathways.

Additionally, variants in *ATP6V0E2*, *DNAH5*, *DNAH6*, *PCDHB7*, *PDE11A*, *TRIOBP*, *TYRP1*, and *WDR81* genes were detected in the present study. However, these eight genes showed no correlations with the investigated pathways related to KC in our study. And there were no studies reported their associations with KC.

Our study has several limitations that should be taken into consideration. Firstly, we only analyzed five KC families in the study due to the limited KC families recruited in our hospital. Secondly, the molecular mechanism of the candidate genes was not explored, and further studies should be performed to explore the mechanism of KC caused by those genes. Thirdly, the putative predisposition variants in noncoding or uncaptured regions of the genome (promoter or intronic variants) were not detectable by exome sequencing.

In conclusion, our family-based exome sequencing studies combined with bioinformatics analysis identified the *EPCAM*, *SHROOM3*, *SYNE1*, *TEK*, and *TTN* genes as potential high-risk candidate pathogenic genes of familial KC. The results obtained significantly improved our understanding of the genetic etiology of the disease, providing novel insights on KC pathogenesis.

## Data Availability

All relevant data were presented in the article. Further inquiries can be directed to the corresponding author.
